# Supplementary material for: Risk factors for sacrococcygeal pilonidal sinus: a systematic review and meta-analysis supplemented by genetic causal assessment
Source: Front Surg. 2026 Jan 7;12:1718589. doi: 10.3389/fsurg.2025.1718589 (PMC12819706; doi:10.3389/fsurg.2025.1718589)
Supplement: Supplementary file 2 [file Datasheet2.zip › Supplementary Data 2/MR_pipeline_p5e-8/leaveone_plots/ebi-a-GCST90018758.finngen_R12_L12_PILONIDALCYST.pdf]

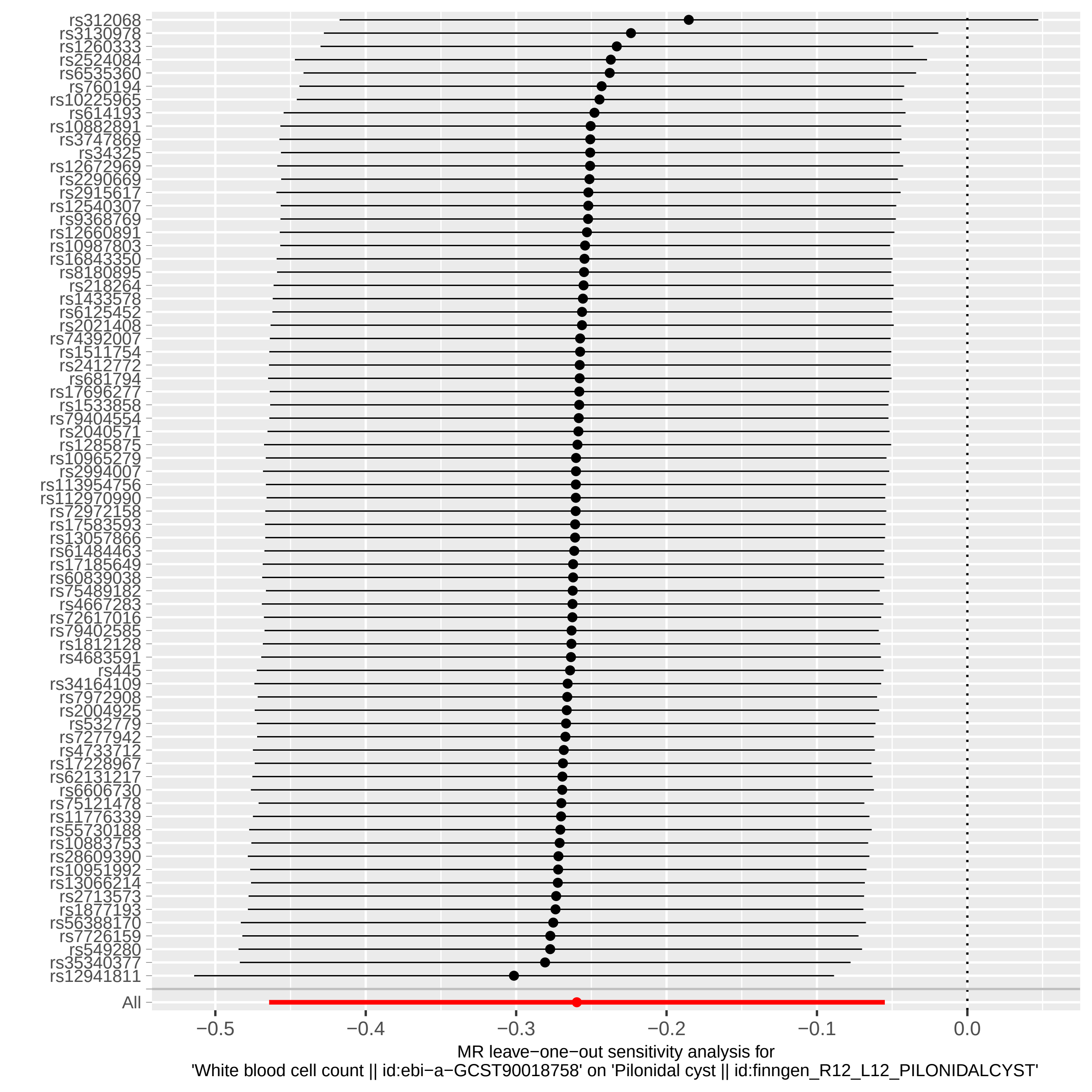
rs312068
rs3130978
rs1260333
rs2524084
rs6535360
rs760194
rs10225965
rs614193
rs10882891
rs3747869
rs34325
rs12672969
rs2290669
rs2915617
rs12540307
rs9368769
rs12660891
rs10987803
rs16843350
rs8180895
rs218264
rs1433578
rs6125452
rs2021408
rs74392007
rs1511754
rs2412772
rs681794
rs17696277
rs1533858
rs79404554
rs2040571
rs1285875
rs10965279
rs2994007
rs113954756
rs112970990
rs72972158
rs17583593
rs13057866
rs61484463
rs17185649
rs60839038
rs75489182
rs4667283
rs72617016
rs79402585
rs1812128
rs4683591
rs445
rs34164109
rs7972908
rs2004925
rs532779
rs7277942
rs4733712
rs17228967
rs62131217
rs6606730
rs75121478
rs11776339
rs55730188
rs10883753
rs28609390
rs10951992
rs13066214
rs2713573
rs1877193
rs56388170
rs7726159
rs549280
rs35340377
rs12941811
All
−0.5
−0.4
−0.3
−0.2
−0.1
0.0
MR leave−one−out sensitivity analysis for
'White blood cell count || id:ebi−a−GCST90018758' on 'Pilonidal cyst || id:finngen_R12_L12_PILONIDALCYST'
